# Effects of Tele-Pilates and Tele-Yoga on Biochemicals, Physical, and Psychological Parameters of Females with Multiple Sclerosis

**DOI:** 10.3390/jcm12041585

**Published:** 2023-02-16

**Authors:** Parisa Najafi, Maryam Hadizadeh, Jadeera Phaik Geok Cheong, Hamidreza Mohafez, Suhailah Abdullah, Maryam Poursadeghfard

**Affiliations:** 1 Faculty of Sports and Exercise Science, Universiti Malaya, Kuala Lumpur 50603, Malaysia; 2 Department of Biomedical Engineering, Faculty of Engineering, Universiti Malaya, Kuala Lumpur 50603, Malaysia; 3 Department of Medicine, Faculty of Medicine, Universiti Malaya, Kuala Lumpur 50603, Malaysia; 4 Clinical Neurology Research Center, Shiraz University of Medical Sciences, Shiraz 7193635899, Iran

**Keywords:** multiple sclerosis, Pilates, yoga, tele-exercise, prolactin, cortisol, mental health, depression, QoL, walking

## Abstract

Background: People with multiple sclerosis (PwMS) suffer from some comorbidities, including physical and psychiatric disorders, low quality of life (QoL), hormonal dysregulation, and hypothalamic-pituitary-adrenal axis dysfunction. The current study aimed to investigate the effects of eight weeks of tele-yoga and tele-Pilates on the serum levels of prolactin and cortisol and selected physical and psychological factors. Methods: Forty-five females with relapsing remitting multiple sclerosis, based on age (18–65), expanded disability status scale (0–5.5), and body mass index (20–32), were randomly assigned to tele-Pilates, tele-yoga, or control groups (*n* = 15). Serum blood samples and validated questionnaires were collected before and after interventions. Results: Following online interventions, there was a significant increase in the serum levels of prolactin (*p* = 0.004) and a significant decrease in cortisol (*p* = 0.04) in the time × group interaction factors. In addition, significant improvements were observed in depression (*p* = 0.001), physical activity levels (*p* < 0.001), QoL (*p* ≤ 0.001), and the speed of walking (*p* < 0.001). Conclusion: Our findings suggest that tele-yoga and tele-Pilates training could be introduced as patient-friendly, non-pharmacological, add-on therapeutic methods for increasing prolactin and decreasing cortisol serum levels and achieving clinically relevant improvements in depression, walking speed, physical activity level, and QoL in female MS patients.

## 1. Introduction

Multiple sclerosis (MS) is a chronic, disabling neurological disorder that reports a 3:1 ratio in the prevalence of females to males [1]. Physical function problems, psychological or mood disturbances, hypothalamic-pituitary-adrenal axis (HPA) dysfunction, and a sexual hormone secretion disorder are all symptoms and signs of MS [2,3]. Although the etiology of MS is unknown [1], genetic factors, hormones, and/or HPA dysfunctions have been implicated in controlling the progression of autoimmune diseases [4,5]. Additionally, sex and stress hormones, including prolactin and cortisol, presumably have complicated mechanisms of function in MS disease [6].

Prolactin has been introduced as a dual-effective hormone in the central nervous system, including repair by supplying regenerative signals to oligodendrocytes, adult neural stem or progenitor cells, and neurons. In contrast, it might increase immune reactions as a harmful effect by stimulating peripheral immune cells [7,8]. Some trials estimated a positive relationship between hyperprolactinemia and MS initiation, relapse, and demyelination [9,10], where the source of this elevation in prolactin is unknown [6]. Furthermore, it has been reported that prolactin levels may increase with the intensity of physical activity [11], including exercise above the anaerobic threshold; however, the initiation of prolactin secretion has been unclear [11]. The prolactin resting level after exercise has shown contradictory results in studies; some showed decreased levels, whereas others reported increased levels [11]. To the authors’ knowledge, only two studies have estimated prolactin levels after resistance training and vibration and yoga training in persons with multiple sclerosis (PwMS), which were reported to increase after intervention [12,13]. As such, this would be a fruitful area for further work.

Cortisol is affected by the hyperactivity of the HPA axis in PwMS, another endogenous product of glucocorticoids that acts as an immunosuppressant and is involved in glucose metabolism and inflammatory and immunological reactions [14,15]. Moreover, cortisol has been introduced as a biomarker of the severity of mental disorders [16]. Some observations concluded that cortisol levels were high and low among PwMS, in which depression and anxiety have been reported with high levels of cortisol in these patients [15,17,18]. The HPA axis is affected by exercise intensity, duration, and rest breaks, with changes in adrenocorticotropic hormone (ACTH) and cortisol levels [19]. Furthermore, for HPA axis stimulation, the exercise intensity threshold must be 50–60% of maximal oxygen absorption (VO2 max) [19]. The highest cortisol reaction has been reported in high-volume and high-intensity interventions, which reduce muscular inflammation, cytokine production, and muscle injury by elevating glucocorticoid sensitivity [20]. According to limited trials published on cortisol level function following exercises in PwMS, Najafi et al. reported a decline in cortisol levels and an enhancement in ACTH levels following yoga exercise [21]. In another study, Sajedi et al. demonstrated that in the MS mouse model, cortisol levels decreased after aerobic exercise combined with probiotic intake [19]. This area needs further study.

Although various styles of interventions have been advised for PwMS as a potential alternative therapy for MS symptom management [2,22], including interval activity [23], resistance exercise [24], aerobic exercise [25], Pilates [23], and yoga [26], lower patient engagement with physical activity has been shown [27,28,29,30,31]. Furthermore, COVID-19, which resulted in many restrictions, increased mental and functional symptoms in PwMS [31]. As a result, home tele-rehabilitation, or tele-health, was recommended as a comparable substitute for symptom prevention in PwMS [32,33]. Yoga and Pilates are popular types of mind–body exercise among the MS community, which have been introduced as rehabilitation in various therapeutic settings such as fatigue, mood, and functional ability [34,35].

There have not been enough comparative studies to examine the effects of tele-yoga and tele-Pilates as user-friendly, cost-effective, and non-instrumental (or requiring simple, accessible devices) on the hormonal secretion levels and the physical and psychological parameters in females with MS. Therefore, in the present 8-week randomized controlled trial (RCT), our primary objective was to evaluate the effects of an online program of yoga and Pilates as complementary non-pharmacological therapy on prolactin and cortisol serum levels and clinically relevant factors such as depression, mental health, QoL, physical activity level, and walking speed in females with MS.

## 2. Methodology

### 2.1. Trial Design

This single-blinded RCT was conducted after the COVID-19 pandemic, when most gyms did not offer exceptional services for these high-risk patients, and these patients still desired to keep social distance. This study was approved by the Medical Research Ethics Committee of Universiti Malaya, Kuala Lumpur, Malaysia (registration number: 2021827-10514) and Shiraz University of Medical Sciences, Shiraz, Iran (registration number: IR.SUMS.MED.REC.1400.586, Iranian Registry of Clinical Trials number: IRCT20211122053145N1).

### 2.2. Participants

Forty-five females with relapsing remitting multiple sclerosis (RRMS) were recruited through the local MS association. A neurologist diagnosed the participants with RRMS according to the diagnosed MS using McDonald’s criteria [36]. The inclusion criteria were: (1) self-reported age (18–65); (2) expanded disability status scale (EDSS) with neurologist diagnosis (0–5.5); and (3) body mass index (BMI) calculated by researchers (20–32). The exclusion criteria were: (1) pregnancies; (2) drug and alcohol addiction; (3) history of cardiovascular disease; (4) abnormal metabolic history; (5) cancer; (6) orthopedic disorders; (7) exercise in the previous three months; (8) relapse in the previous three months. The participants from the Shiraz MS Center, Iran, were recruited through the dissemination of posters and participation information leaflets on social media and text messages to members of the MS society. The researchers contacted interested participants through video calls and explained every step of the research process. Before starting the protocol, the participants signed a written informed consent form.

### 2.3. Intervention

The online supervised interventions lasted eight weeks, three times a week (24 sessions), in each tele-yoga and tele-Pilates exercise group, whereas the control group did not implement an intervention. Each session included three phases: warming up (10–15 min), main poses (30–40 min), and cooldown (10–15 min). In addition, three user-friendly online visual platforms were used, including Google Meet, Zoom, and Instagram. Furthermore, belts, blocks, chairs, walls, and resistance bands were used as modifier equipment for various postures.

#### 2.3.1. Tele-Yoga

A certified yoga instructor with 12 years of experience with PwMS supervised the sessions. The Hatha yoga program was slightly modified to meet the needs and restrictions of PwMS. Hatha yoga is a popular type of yoga that incorporates postures (asanas), meditation (Dyana), and breathing methods (pranayama). Each pose was held for 10 to 30 s, followed by a 30 s to 1 m of relaxation between asanas. Every 60 min session was followed by a 10–15 min warm-up with pawanmuktasanas, 30–40 min main movements, and a 10–15 min cooldown, which included a deep relaxation period in which participants lay supine (corpse pose), meditation, and breathing methods. The difficulty in movements and the duration of the stay in each pose were altered among participants to provide progression.

#### 2.3.2. Tele-Pilates

Tele-Pilates was implemented for 60 min per session by a Pilates professional trainer. Warm-up exercises lasted 10–15 min, main body movements were 30–40 min long, and cooldown activities lasted 10–15 min. All of the exercises were performed while adhering to neutral spine alignment, voluntary deep abdominal muscular activation, and combined breathing in various postures (prone, sitting, supine, 4-point kneeling, side-lying, and standing). The exercises were developed by changing the support base from level 1 to level 3 positions.

#### 2.3.3. Control Min

The control group of female MS patients maintained their current lifestyle without regular physical activity or exercise for eight weeks. The participants were asked to perform a blood and walking test and fill out four questionnaires before and after eight weeks of no intervention.

### 2.4. Outcomes

The primary outcomes were cortisol and prolactin serum levels, walking speed, depression, mental health, QoL, and physical activity. Experts collected blood samples, walking speeds, and other factors through self-reported questionnaires 24 to 48 h before and after the interventions. All the questionnaires used in this study, including the Multiple Sclerosis Quality of Life (MSQoL-54), General Health Questionnaire (GHQ-12), the International Physical Activity Questionnaire (IPAQ), and the Beck Depression Inventory (IBD), were reported as reliable and valid measurements for PwMS and were also translated into the Persian language [37,38,39,40,41].

#### 2.4.1. Blood Samples Analysis

The cortisol and prolactin serum levels were taken 24–48 h before and after the training session. Blood samples (25 mL) were centrifuged for 10 min at 3000 g, and the serum was aliquoted and refrigerated at −80 °C. After collecting all of the samples, the samples were analyzed using the ELISA technique. Cortisol and prolactin were measured by using ELISA kits (Lake Forest, California 92630, USA) and (PishtazTeb Diagnostics, Tehran, Iran), respectively. Of note, the clinical evaluations of serum blood tests were performed when most of the female MS participants were in the follicular phase to avoid the error variation of prolactin that may occur in the menstrual cycle [42]. Prolactin blood was collected three to four hours after awakening [43,44,45], and the cortisol serum level was not exceeded by 8:30 a.m. [36,46,47]. Furthermore, the participants were informed that they should not drink caffeine or perform heavy physical activity on the day of and the day before blood testing [47].

#### 2.4.2. Depression

The Beck depression inventory (BDI) is one of the most widely used psychometric tests for determining the degree of depression, particularly in PwMS [48]. In our research, depression was assessed with Beck’s 21-question multiple-choice self-report questionnaire, which categorized depression into six levels between one and forty: ups and downs are considered normal (1–10), mild mood disturbance (11–16), borderline clinical depression (17–20), moderate depression (21–30), severe depression (31–40), and extreme depression over 40 [49], where low scores indicate a better level of depression.

#### 2.4.3. Mental Health

The psychological state of mind will be evaluated with the General Health Questionnaire (GHQ), translated into 38 languages since its development, and indicate its formal validity across cultures [50]. The 12-item GHQ is a self-administered screening tool to detect current-state mental disturbances and disorders in a primary care setting. It uses a 4-point Likert-type scale (0 to 3), with a total score that could range between 0 and 36 [51]. The mental health levels were classified as perfect (0–6), good (7–12), fairly good (13–18), not bad (19–24), bad (25–30), and extremely bad (31–36) [52], where lower scores showed a better level of mental health.

#### 2.4.4. Physical Activity Levels

The fastest and most cost-effective way to measure a large population’s physical activity level is by assessing it through the international physical activity questionnaire (IPAQ). The short IPAQ form (which assesses the last 7 days) is advised for national monitoring, and the long form for research needs a more detailed evaluation. Work-related, transportation-related, household, and leisure-time physical activity are among the various domains covered by the IPAQ form [53]. These factors were computed to the metabolic equivalent of task (MET) minutes per/week by reproducing the exercise level (3.3, 4, or 8) in minutes and the number of days per week [54]. The levels of physical activity are divided into three levels: vigorous physical activity = total MET-min/week> 1500; moderate physical activity = 480 ≤ total MET-min/week ≤ 1500; and passive (inactive) = total MET-min/week ≤480 [54]. Each level contains four sub-scores: vigorous activity, moderate activity, walking activity, and sitting time. The activity level was assessed by adding the scores of the three vigorous, moderate, and walking activities.

#### 2.4.5. Timed 25-Foot Walk (T25FW)

The T25FW test was carried out on a 7.62-m-long, clearly defined path, on which participants walked as quickly and safely as possible three times, and the average of their times was recorded. It has been determined that the T25FW test is valid and reliable for PwMS [35], and the low scores over time have shown improvement in the walking speed of PwMS.

#### 2.4.6. Quality of Life (QoL)

The results from the international quality of life study on multiple sclerosis (MSQoL-54) scores obtain two separate outputs: (1) the physical health composite score, which necessitates eight questionnaire parts: physical role limitations, pain, health perceptions, sexual function, social function, physical function, health distress, and energy or fatigue; and (2) the mental health composite score, which consists of five questionnaire parts: overall QoL satisfaction, emotional well-being, and emotional and cognitive function limitations. The physical and mental health composite scores were assessed with 12 scales among the 52 elements of an MSQoL-54 questionnaire (ranging from 0 to 100). As such, higher values in these two dimensions have shown better QoL levels [55,56,57].

### 2.5. Sample Size

Previous studies employed different outcome variables and statistical analyses than the current study. As such, the random effect size was estimated using Cohen’s conventions [58] for F-test family statistical analysis (“small,” “medium,” and “large” as 0.1, 0.25, and 0.4, respectively). G-power software (3.1.9.7, written by Franz Faul, Universität Kiel, Germany) was used to calculate the sample size needed for a mixed-design ANOVA with a medium effect size = 0.25, α = 0.05, and power = 0.8 [59], number of groups = 3, number of measurements = 2, yielding a total sample size of 42. After accounting for the 10% dropout, we need to recruit 45 participants for our study to achieve 15 participants per group. 

### 2.6. Randomization

The participants were randomly assigned to one of three groups: control, tele-yoga exercise, or tele-Pilates exercise. Researchers who did not participate in independent outcome evaluations used simple randomization procedures (www.randomizer.org, accessed on 15 March 2022) for randomization. The participants were notified of allocation by the lead author.

### 2.7. Data Analysis

Statistical analyses were performed using SPSS for Windows, version 26. To determine whether tele-exercise affects cortisol and prolactin serum levels, depression, QoL, mental health, physical activity level, and walking speed, we used a mixed-factor ANOVA (between factor—3 groups: tele-yoga, tele-Pilates, and control; within factor—2 times: pre- and post-intervention), and the preliminary assumptions were checked without serious violations [60,61]. Normality, as one of the assumptions of the dependent variables, was tested for each cell of the design (3 groups x 2 measurements = 6 cells) using both numerical (Shapiro–Wilk test of normality, skewness, and kurtosis values) and graphical (histograms and Q-Q plot) methods. The data were presented as mean ± standard deviation (SD). If the time-group interaction showed significant changes, post hoc tests (Bonferroni) for time factor changes were performed from repeated measures tests after splitting into three groups, and univariate tests were performed for group factor effects. The significance level was set at *p* < 0.05 in all tests.

## 3. Results

### 3.1. Characteristics of Feasibility

**Figure 1:**
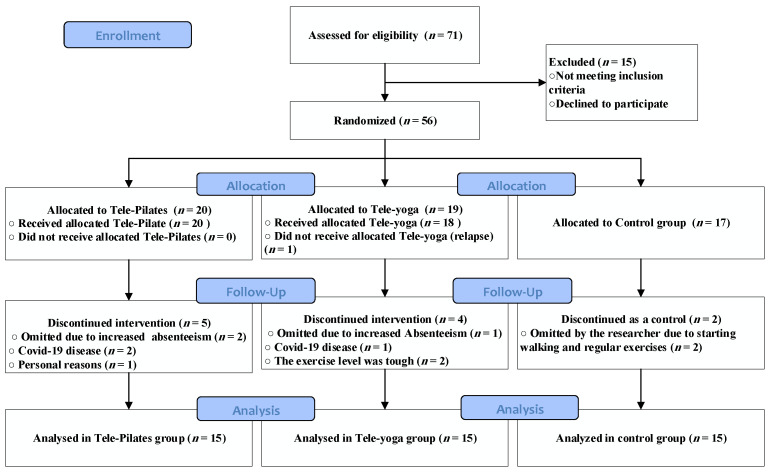
Participants’ flow in an 8-week tele-exercising program.

Out of 71 participants, 56 females with RRMS were included in the study following the inclusion criteria. Figure 1 illustrates the participants’ flow in an 8-week experimental design. Meetings with patients were conducted in groups, and the attendance ratio was recorded in both groups. Eventually, some absences were covered by a tele-training compensation session. As such, 45 participants continued in three equal groups (*n* = 15), including age: (38.00 ± 5.46), BMI: (23.06 ± 1.30), and EDSS: (2.55 ± 1.16): tele-Pilates, tele-yoga exercise, and control patients with MS group. Table 1 summarizes the demographic characteristics of our participants. In addition, the participants were using Fingolimod, β-Interferon, Rituximab, Ocrelizumab, and Natalizumab as disease-modifying treatment (DMT).

### 3.2. Outcomes and Estimations

The results of the mean and *p*-value of the variables’ analysis are summarized in Table 2. All variables except mental health have reported significant changes in the time × group interaction factor (all *p* < 0.05).

#### 3.2.1. Resting and Post-Exercise Serum Prolactin Levels

**Figure 2:**
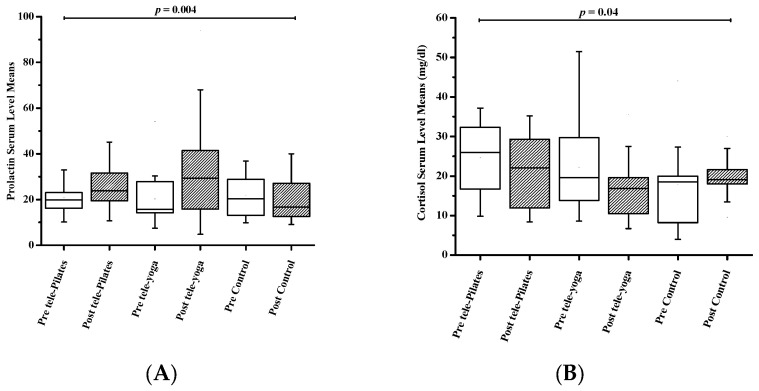

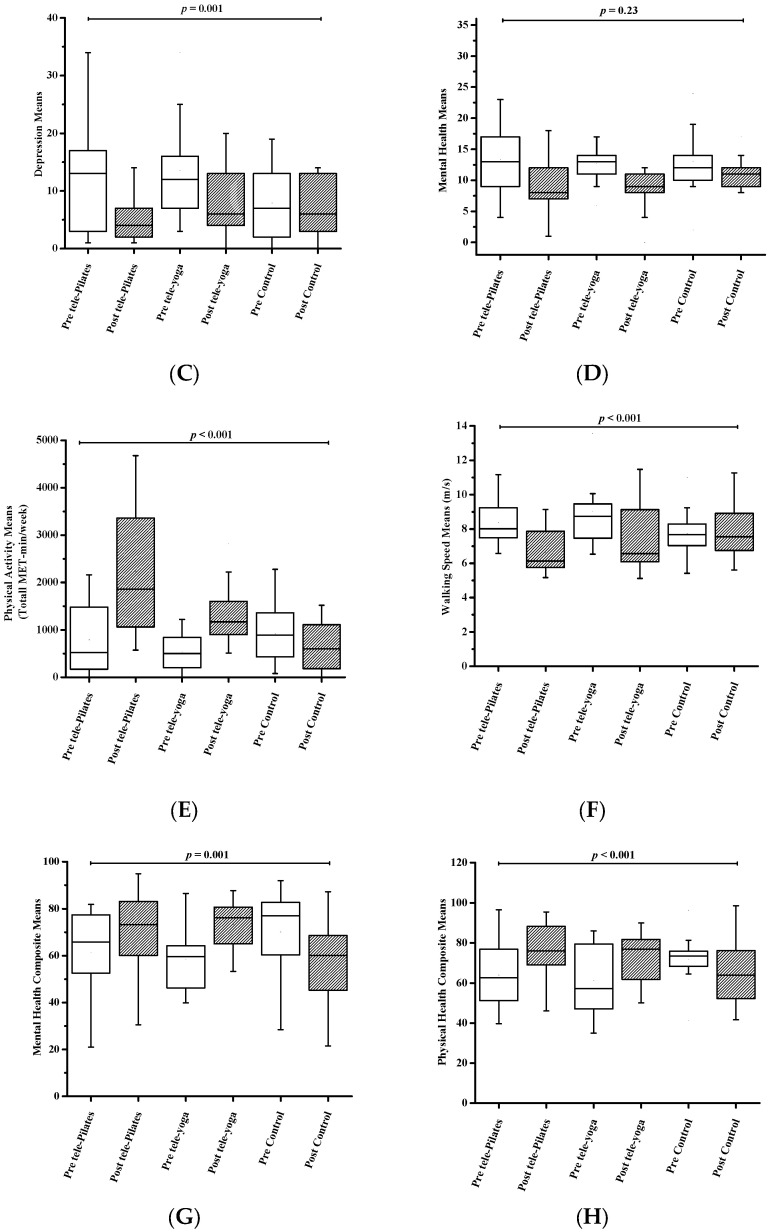
Changes in variables between groups during the 8-week period. Statistically significant (*p* < 0.05); (**A**) prolactin serum level (ng/mL); (**B**) cortisol serum level (mcg/dL); (**C**) beak depression introvertly (IBD) score: 1 ≤ these ups and downs are considered normal ≤ 10; 11 ≤ mild mood disturbance ≤ 16; 17 ≤ borderline clinical depression ≤ 20; 21 ≤ moderate depression ≤ 30; 31 ≤ moderate depression ≤ 40; 31 ≤ severe depression ≤ 40; over 40 extreme depression; (**D**) general health questioner (GHQ-12) score = 0 ≤ perfect ≤ 6; 7 ≤ good ≤ 12; 13 ≤ fairly good ≤ 18; 19 ≤ not bad ≤ 24; 25 ≤ bad ≤ 30; 31 ≤ extremely bad ≤ 36; (**E**) international physical activity (IPAQ) score: vigorous physical activity = total MET-min/week > 1500; moderate physical activity = 4 80 ≤ total MET-min/week ≤ 1500; passive(inactive) = total MET-min/week ≤ 480; (**F**) speed of walking (T25FW) (s); (**G**) mental health composite score; (**H**) physical health composite score.

In prolactin serum levels, a significant increase in time factor (F_1,42_ = 11.65, *p* = 0.001) and factor of time × group interaction (F_2,42_ = 6.25, *p* = 0.004) was reported, but group factor has not shown significant changes (F_2,42_ = 0.79, *p* = 0.46). As a result, the post hoc test revealed an increase in serum prolactin levels following an 8-week tele-yoga (*p* = 0.01, η^2^ = 0.39) and tele-Pilates (*p* = 0.01, η^2^ = 0.40) program. The breakdown of these results is shown in Table 2 and Figure 2.

#### 3.2.2. Resting and Post-Exercise Serum Cortisol Levels

The interaction between the time × group (F_2,42_ = 3.46, *p* = 0.04) and the time factor (F_1,42_ = 4.46, *p* = 0.04) has significantly decreased serum levels of cortisol. However, the group factor (F _2,42_ = 1.11, *p* = 0.34) did not report a significant difference. Table 2 and Figure 2 illustrate the breakdown of cortisol serum levels. Furthermore, the post hoc test showed a decrease in cortisol serum levels after tele-Pilates (*p* = 0.002, η^2^ = 0.51) and tele-yoga (*p* = 0.02, η^2^ = 0.33) exercising.

#### 3.2.3. Depression

Depression was estimated with scores from the Beck questionnaire, which are presented in Table 2 and Figure 2. Depression declined significantly in the time × group interaction (F_2,42_ = 8.94, *p =* 0.001) and the time factor (F_1,42_ = 25.72, *p* < 0.001) following tele-exercising, but the factor of group (F_2,42_ = 1.46, *p =* 0.24) has not illustrated significant changes. The post hoc test following tele-Pilates and tele-yoga reported (*p* < 0.001, η^2^ = 0.74) and (*p* = 0.001, η^2^ = 0.55), respectively.

#### 3.2.4. Mental Health

Mental health, which did not reveal significant changes over the time × group interaction factor (F_2,42_ = 1.50, *p* = 0.23) or the group factor (F_2,42_ = 0.99, *p* = 0.38), but did for the time factor (F_1,42_ = 24.57, *p* < 0.001), has shown improvement. Moreover, post hoc comparisons indicated that mental health improved in tele-yoga (*p* < 0.001, η^2^ = 0.63) and tele-Pilates (*p* < 0.001, η^2^ = 0.68) the groups post-test (Figure 2, Table 2).

#### 3.2.5. Physical Activity

In terms of physical activity level, a significant enhancement was shown in the time × group interaction factor (F_2,42_ = 12.39, *p* < 0.001), group factor (F_2,42_ = 6.22, *p* = 0.004), and time (F_1,42_ = 20.23, *p* < 0.001). Post hoc analysis showed the improvement in physical activity following tele-Pilates (*p* = 0.002, η^2^ = 0.52) and tele-yoga (*p* < 0.001, η^2^ = 0.68) exercising. The results obtained from the preliminary analysis of the physical activity levels are shown in Table 2 and Figure 2. Additional sub-scores of each level for physical activity have been shown in Table 3. Vigorous activity has reported a significant enhancement in the group × time factor (F_2,42_ = 4.38, *p* = 0.02), but there were no significant changes in the time (*p* = 0.32) or group factors (*p* = 0.59) after interventions. Furthermore, the time × group interaction (F_2,42_ = 7.34, *p* = 0.002) showed significant improvements in moderate activity, but the group (*p* = 0.25) and time (*p* = 0.07) factors did not show significant differences after interventions. Of note, walking activity has shown significant improvement in time × group interaction, group, and time factors after tele-exercising (all *p* < 0.001).

#### 3.2.6. Timed 25-Foot Walk

The speed of walking was significantly increased with time (F_1,42_ = 43.79, *p* < 0.001) and interaction between time × group factors (F_2,42_ = 14.86, *p* < 0.001). Yet, the group factor did not show significant changes (F_2,42_ = 0.99, *p* = 0.38). In addition, post hoc tests indicated improvements in walking speed after the tele-exercising Pilates and yoga groups, (*p* < 0.001, η^2^ = 0.83) and (*p* < 0.001, η^2^ = 0.65), respectively. The results obtained from the analysis of T25FW scores are summarized in Table 2 and Figure 2.

#### 3.2.7. Quality of Life (QoL)

Physical and mental health composite scores that demonstrate QoL are shown in Table 2 and Figure 2. Of note, in the interaction between time × group (F_2,42_ = 8.90, *p* = 0.001), the time (F_1,42_ = 12.49, *p* = 0.001) factors reported significant improvements in the mental health composite, but the group factors (F_2,42_ = 0.14, *p* = 0.87) did not change significantly. The post hoc comparisons reported significant enhancement following tele-yoga (*p* < 0.001, η^2^ = 0.64) and tele-Pilates (*p* < 0.001, η^2^ = 0.65) exercises. Furthermore, although the group factor (F_2,42_ = 0.15, *p* = 0.90) did not show significant differences, the physical health composite improved significantly in the time × group interaction (F_2,42_ = 9.56, *p* < 0.001) and the time (F_1,42_ = 7.42, *p* = 0.01) factors. Post hoc analysis revealed an increase in the physical health composite factor after tele-Pilates (*p* = 0.01, η^2^ = 0.36) and tele-yoga (*p* = 0.01, η^2^ = 0.43), and a decrease in the control group (*p* = 0.02, η^2^ = 0.35).

## 4. Discussion

Our research is one of the first studies that shows the feasibility of Pilates and yoga interventions as online exercises on biochemical parameters such as cortisol and prolactin, physical parameters such as walking speed, and psychological parameters such as mental health, depression, and QoL in females with MS.

HPA dysfunction and hormonal dysregulation are the symptoms of patients with MS [21]. The results show that prolactin serum levels increased after 8 weeks of intervention in the tele-yoga and tele-Pilates groups. The enhancement showed more changes after tele-yoga (32.62 ± 23.84) compared with tele-Pilates (25.83 ± 8.66) but was not statistically significant. This study supports evidence from Avandi (2017), who showed an increase in prolactin after yoga exercises [13]. Another study reported that exercise could enhance prolactin levels, and this enhancement also depends on the intensity and duration of the intervention [62]. As prolactin plays a dual role in MS immune pathogenesis, it influences CD40 expression in B cells and increases B cell autoreactivity in MS, which may demonstrate a link between brain injury and prolactin levels by enhancing myelin regeneration and neuronal survival in MS animal models. [43]. As such, a possible explanation for our results might be that tele-Pilates and tele-yoga may help female patients with MS to repair their myelin by increasing prolactin serum levels [43,63]. In contrast, it has been indicated that an increase in prolactin levels may also raise inflammatory reactions and injure MS patients [43,64]. Eventually, to develop a full picture of prolactin’s role in MS patients after exercise, additional studies will be needed.

Another important finding was that a significantly decreased cortisol level was reported after tele-Pilates and tele-yoga in females with MS. The decreases in cortisol serum levels after tele-yoga (−5.94 ± 2.25) were more significant than those after tele-Pilates (−3.56 ± 0.94), and this difference was not statistically significant. These results agree with our previous finding by Najafi et al. (2017), showing a decline in cortisol serum levels after yoga exercise in MS patients [21]. Although Sajadi et al. (2021) support the idea of a decreased cortisol level after aerobic exercise in patients with MS, they have not significantly decreased [19]. Indeed, high cortisol levels in MS patients can be caused by HPA axis dysfunction [21], which leads to clinically relevant depression, mental disorders, anxiety, and disease progression [15,17,65]. As such, a possible explanation may be a decrease in cortisol levels due to the HPA axis being modulated following tele-yoga and tele-Pilates training.

Following the relationship between cortisol and psychiatric symptoms [15], the participants’ depression and mental health levels were assessed by questionnaires. According to our findings, depression improved after tele-exercising, with tele-yoga (−8.07 ± 1.97) showing a significant decrease in depression scores vs. tele-Pilates (−4.26 ± 0.67), but this difference was not significant. Indeed, depression levels statistically changed from “mild mood disturbance” to the “these ups and downs are considered normal” level following interventions. This finding was consistent with previous research that found yoga and home-based Pilates have a positive effect on depression in PwMS [66,67], and this agreement is reflected in a systematic review that evaluated 40 studies after Pilates intervention [68]. Furthermore, after tele-intervention, mental health showed an improvement trend, but it was not statistically significant. Indeed, tele-yoga and tele-Pilates as mind–body exercises may play a dual role in depression and mental health improvement among female patients with MS. Tele-yoga and tele-Pilates may have caused clinical improvements in psychiatric factors by modulating HPA axis dysfunction [69] and reducing cortisol levels, or vice versa. The greater improvement in mental symptoms after tele-yoga may partly be explained by the special mind exercises of yoga, such as meditation, pranayama, and savasana, which can affect the mind’s calmness, anxiety, and stress relief [69].

Another notable finding from our study was a significant increase in physical activity levels from moderate to vigorous following tele-Pilates, and from inactive to moderate following tele-yoga exercises in females with MS. Our interventions only resulted in an increase of 480 MET min/week [54]. In contrast, the mean changes in physical activity improvement after both tele-exercises were greater. This study supports evidence from previous findings [70,71]. Observations were taken after physical activity, and the T25FW test showed improvements in walking speed following tele-yoga and tele-Pilates in females with MS. These results are in agreement with Abasıyanık et al. (2020) and Kalron et al.’s (2016) findings [35,72]. In our study, a possible explanation for the improvements in physical activity and walking speed might be that tele-yoga may help by altering various factors such as reducing stress or tension in muscles, improving mobility, blood circulation, and flexibility in joints and tendons in female patients with MS, and also that the tele-Pilates exercise protocol improves functional factors such as balance, strength, and stability to enhance muscle capability and tolerability. In addition, improving muscle control through appropriate intra- and intermuscular contractions appears to be one of the major determinants of reduced walking speed in PwMS [73].

In the current study, physical and mental health composites, which indicated QoL, improved following tele-yoga and tele-Pilates in female patients with MS. Furthermore, the mental health composite showed more changes in tele-yoga exercise (14.91 ± 2.21) than in tele-Pilates training (9.92 ± 2.23) in females with MS. These findings appear to be consistent with other studies that used Pilates and yoga [74,75]. In our research, increased QoL following tele-yoga may be caused by focusing on positive thoughts with deep breathing, reducing stress levels, and improving mobility and flexibility through asanas [76]. In addition, tele-Pilates may be a sort of active meditation that challenges participants to focus on the activation of deep abdominal muscles and posture with breathing, which may affect QoL [35].

One of the key strengths of this RCT is that the constraints on going to the gym and rehabilitation centers were partially resolved for these patients, who have limitations due to their bladder and physical function problems. In addition, we made the homes of this high-risk group for contracting COVID-19 a safer place to exercise. We used free visual platforms for internet-based exercise and created a space for more individuals to participate from wider areas. On the other hand, our intervention period was limited, a longer duration for future tele-intervention will be recommended. Our study only assessed the effects of tele-exercise on females with RRMS; therefore, its results might not be generalizable to patients with other types of MS, such as PPMS and SPMS, or another gender.

## 5. Conclusions

The investigation results after eight weeks of tele-Pilates and tele-yoga in females with MS showed an increase in prolactin serum levels, which may help myelin repairment; a decrease in cortisol that may be caused by adjusting HPA dysregulation; improvements in depression that may be due to reductions in cortisol serum levels, or vice versa; and the enhancement of physical activity levels, walking speed, and QoL. Our findings suggest the feasibility of tele-yoga and tele-Pilates as non-pharmacological add-on therapies that may have a beneficial impact on hormonal secretion and physical and mental health factors in females with MS.

## Figures and Tables

**Table 1:** Demographic characteristics of female participants.

	Tele-Pilates Group (*n* = 15)	Tele-Yoga Group (*n* = 15)	Control Group (*n* = 15)	Total (*n* = 45)
Age (year)	36.20 ± 4.33	37.40 ± 6.03	40.40 ± 5.35	38.00 ± 5.46
EDSS	2.5 ± 1.32	2.5 ± 1.19	2.66 ± 1.03	2.55 ± 1.16
BMI (kg/m^2^)	22.14 ± 1.27	23.06 ± 1.07	23.99 ± 0.82	23.06 ± 1.30
EMPL/UEMPL	4/11	4/11	6/9	14/31
Dip/UE	6/9	7/8	4/11	17/28
MDO (year)	10.93 ± 4.38	8.00 ± 5.84	9.27 ± 8.37	9.40 ± 5.61

Note: Values are presented as mean ± standard deviation (SD), EDSS: Expanded disability status scale; m: meter: kilogram; BMI: body mass index. Dip: diploma; UE: university education; EMPL: Employed; UEMPL: unemployed; MDO: mean year of disease onset.

**Table 2:** Effects of 8 weeks of tele-exercising in measurement scores.

	Tele-Pilates Exercising (*n* = 15)		Tele-Yoga Exercising (*n* = 15)		Control Group (*n* = 15)		(*p*-Value)		
	Pre-Intervention	Post-Intervention	Pre-Intervention	Post-Intervention	Pre-Intervention	Post-Intervention	Time	Group	Time Group *
Prolactin (ng/mL)	20.79 ± 6.78	25.83 ± 8.66	20.33 ± 11.72	32.62 ± 23.84	21.74 ± 9.16	20.46 ± 9.08	0.001 *	0.46	0.004 *
Cortisol (mcg/dL)	24.62 ± 8.86	21.06 ± 8.81	22.26 ± 12.09	16.31 ± 7.84	17.86 ± 2.68	19.62 ± 5.78	0.04 *	0.34	0.04 *
BDI scores	13.00 ± 9.46	8.73 ± 8.25	13.60 ± 8.37	5.53 ± 4.71	7.93 ± 6.27	6.87 ± 4.91	<0.001 *	0.24	0.001 *
GHQ-12 scores	13.33 ± 5.03	9.31 ± 4.37	12.67 ± 2.99	8.07 ± 3.80	13.07 ± 5.62	11.26 ± 2.89	<0.001 *	0.38	0.23
IPAQ scores (MET-min/week)	790.47 ± 707.26	2230.00 ± 1351.60	535.60 ± 381.27	1296.00 ± 636.79	917.27 ± 651.68	636.67 ± 505.60	<0.001 *	0.004 *	<0.001 *
T25FW (m/s)	8.36 ± 1.25	6.62 ± 1.21	9.02 ± 2.04	7.49 ± 2.02	7.75 ± 1.32	7.91 ± 1.44	<0.001 *	0.38	<0.001 *
MSQoL-54 Scores									
MHC scores	61.45 ± 17.99	71.37 ± 16.90	58.34 ± 13.69	73.25 ± 9.97	70.10 ± 17.51	57.39 ± 19.59	0.001 *	0.87	0.001 *
PHC scores	63.93 ± 17.44	74.42 ± 14.51	61.16 ± 16.86	72.72 ± 12.69	71.56 ± 12.22	65.02 ± 14.60	0.01 *	0.90	<0.001 *

Note: Data are presented as mean ± standard deviation (SD); *: Refers to level of significance (*p* < 0.05) in the analysis of repeated measures ANOVA; m: meter; s: second; mcg/dl: micrograms per decalitre; ng/mL: nanogram per milliliters; BDI: beak depression inventory; GHQ-12: general health questioner; IPAQ: international physical activity questioner; MSQoL-54: multiple sclerosis quality of life-54; MHC: mental health composite; PHC: physical health composite.

**Table 3:** The discrepancies of activities of each physical activity level between the groups.

	Tele-Pilates Exercising		Tele-yoga Exercising		Control Group		(*p*-Value)		
	Pre-Intervention	Post-Intervention	Pre-Intervention	Post-Intervention	Pre-Intervention	Post-Intervention	Time	Group	Time Group *
Vigorous activity	161.13 ± 112.27	610.67 ± 235.92	58.67 ± 31.68	322.67 ± 129.85	472.00 ± 284.19	109.33 ± 46.59	0.32	0.59	0.02 *
Moderate activity	466.67 ± 157.98	822.67 ± 86.34	351.47 ± 75.25	720.00 ± 85.05	561.33 ± 161.59	282.67 ± 92.55	0.07	0.25	0.002 *
Walking activity	162.67 ± 33.42	796.67 ± 152.86	125.47 ± 31.78	253.33 ± 56.35	306.27 ± 60.26	244.67 ± 68.28	<0.001 *	<0.001 *	<0.001 *

Note: Data are presented as mean ± standard deviation (SD); * refers to the level of significance (*p* < 0.05) in the analysis of repeated measures ANOVA.

## Data Availability

The data that support the findings of this study are available from the corresponding author upon reasonable request.
